# Evaluating the Impact of Cartoon-Based Learning on Student Performance and Engagement in Medical Education: An Experimental Study

**DOI:** 10.7759/cureus.54684

**Published:** 2024-02-22

**Authors:** Rajeswari Kathiah, Praveena Daya A, Saraswathy MP, Sathish Selvakumar

**Affiliations:** 1 Pathology, All India Institute of Medical Sciences, Madurai, Madurai, IND; 2 Community and Family Medicine, All India Institute of Medical Sciences, Madurai, Madurai, IND; 3 Microbiology, Employees' State Insurance Corporation (ESIC) Medical College and Post Graduate Institute of Medical Science and Research (PGIMSR), Chennai, IND; 4 Pathology, Employees' State Insurance Corporation (ESIC) Medical College and Post Graduate Institute of Medical Science and Research (PGIMSR), Chennai, IND

**Keywords:** curriculum enhancement, medical student engagement, supplementary teaching techniques, visual learning aids, randomized control trial, student learning outcomes, innovative educational tools, medical teaching methods, cartoon-based learning, pathology education

## Abstract

Background: Pathology, a foundational yet challenging subject in medical education, is marked by its extensive content and intricate concepts. These complexities often pose a significant learning barrier for students, who must not only comprehend but also effectively apply this knowledge in their clinical practice.

Objective: This study aimed to investigate the impact of utilizing cartoons as a supplementary educational tool in pathology. Specifically, it focused on assessing whether incorporating cartoons into the learning process would enhance students' understanding, memory retention, and ability to recall complex topics, thereby augmenting the effectiveness of traditional teaching methodologies.

Materials and methods: Conducted from June to September 2022, this experimental study involved 106 second-year MBBS (Bachelor of Medicine and Bachelor of Surgery) students. Participants were split into two groups: the "traditional group," which received standard interactive large-group teaching, and the "combination group," which benefited from both the standard teaching and additional cartoon-based instruction. The study focused on two selected chapters of the pathology curriculum. After completing the first chapter, the groups were crossed over for the second chapter. Evaluation of the students' learning was conducted through post-learning assessments using multiple-choice questions (MCQs).

Results: The combination group, which received both traditional and cartoon-based teaching, showed a significant improvement in their assessment scores compared to the traditional group. This improvement was consistent in both assessments conducted (t(102) = 8.41, p < .001 and t(99) = 6.85, p < .001). Additionally, feedback from the students through a post-learning survey indicated a strong preference for the use of cartoons. The majority of students agreed that cartoons facilitated a better understanding and retention of complex pathology topics (χ² = 130.9, p < 0.001).

Conclusion: The incorporation of cartoons as a supplementary learning tool in pathology teaching shows promising results. This innovative approach not only complements but also enhances the traditional teaching methods, leading to improved comprehension, retention, and recollection of complex subjects among medical students. The study highlights the potential of cartoons in revolutionizing medical education, especially in teaching challenging subjects like pathology.

## Introduction

The landscape of medical education is continually transforming, particularly in the recent decade, reflecting significant advancements and innovations. Education, inherently dynamic, demands regular refinement and evolution. The absence of creative and innovative teaching methodologies risks rendering medical curricula obsolete, and failing to adequately prepare future medical professionals. Consequently, contemporary shifts in medical education emphasize the imperative to adopt and integrate novel teaching and learning methods. These methods are tailored to meet the expectations and learning styles of the new generation of medical students. Among these modern methodologies are diverse approaches like case-based learning, evidence-based medicine, problem-based learning, simulation-based learning, e-learning, peer-assisted learning, observational learning, the flipped classroom concept, and team-based learning. Each of these methodologies plays a crucial role in enriching and diversifying the medical education experience [1,2].

The objectives of the study are (1) to assess the effectiveness of cartoons in enriching students' comprehension of complex pathology topics when integrated with conventional teaching approaches; (2) to investigate the efficacy of cartoons as a tool in enhancing students' ability to recall and apply complex subject matter in assessments; and (3) to quantify the impact of cartoons as a supplementary teaching tool through student feedback and to establish if there is a statistically significant preference for this method of learning among medical students.

## Materials and methods

Study design

**Figure 1:**
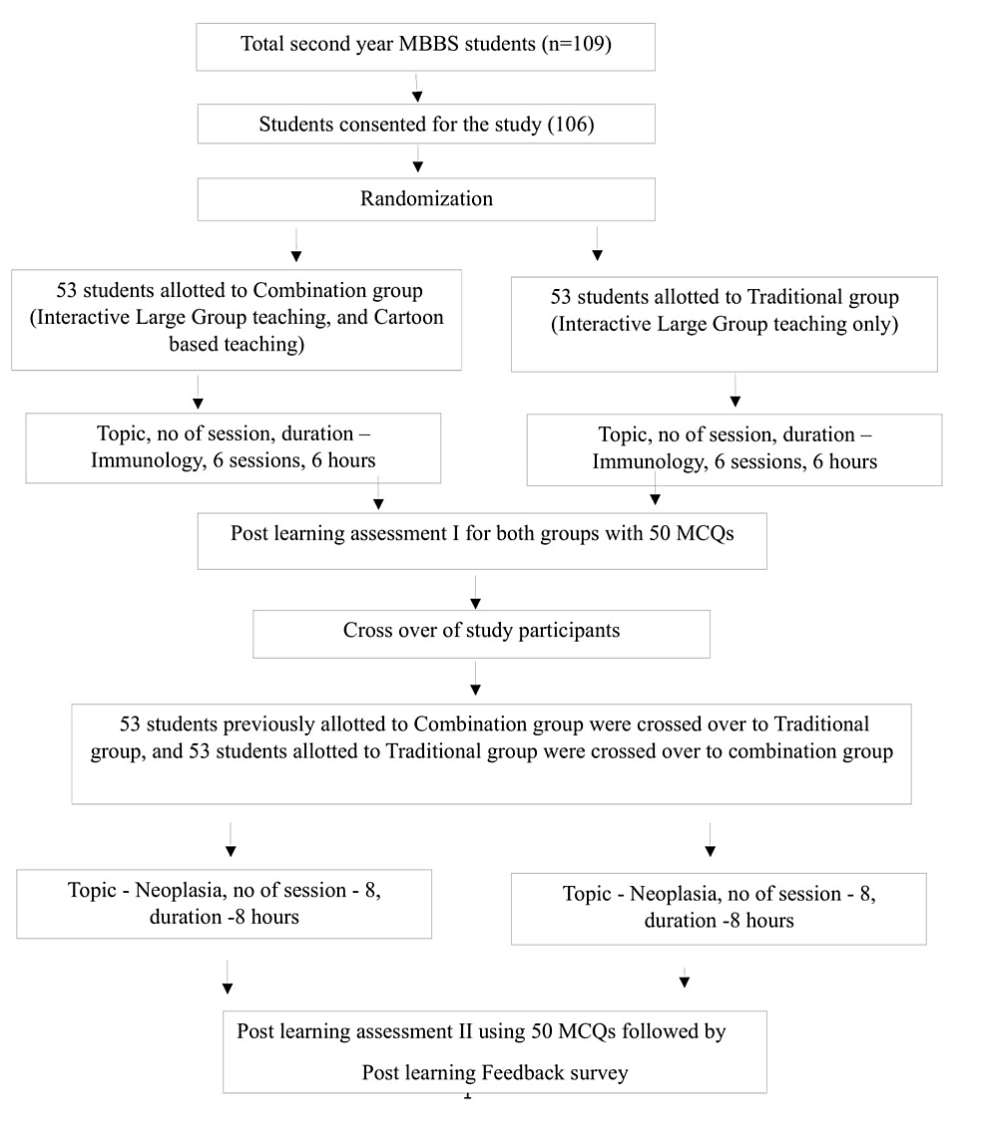
Flow of study participants into the study, and the process of the study MBBS: Bachelor of Medicine and Bachelor of Surgery; MCQ: Multiple choice questions

This experimental study was conducted from June to September 2022, involving second-year MBBS (Bachelor of Medicine and Bachelor of Surgery) students. After receiving approval from the institutional ethics committee and obtaining consent from participants, the study commenced. Purposive sampling was employed, and 106 out of 109 students consented and were included. However, due to absences in one or both assessments, the final study comprised 103 students (Figure 1).

Sample size calculation

A preliminary pilot trial with 25 third-year medical students, not included in the main study, informed the sample size calculation. Utilizing Open Epi software version 3.0 (Open Source Epidemiologic Statistics for Public Health, www.OpenEpi.com) and based on the pilot's mean ± SD scores, a sample size of 106 (53 students per group) was determined to detect a 3% difference in post-learning test scores between groups, with a 2-sided type I error rate of 0.05 and 80% power.

Study materials

The chosen chapters for instruction were Immunology and Neoplasia. These chapters were selected for instruction because they are recognized as challenging chapters within the realm of General Pathology. To ensure impartial outcomes, the same faculty member conducted all sessions for both groups, albeit in separate sessions. The chapters and the chosen topics are listed in Table 1.

**Table 1:** Chapters and topics chosen for the study

Chapter I: Immunology duration of teaching: One month (July 2022); Number of sessions: 6	Topics: Immune response, Immunological tolerance, Hypersensitivity reactions, Primary immunodeficiency, Leukocyte adhesion deficiency type 1 & 2, Pathogenesis of HIV; Number of cartoons: 7
Chapter II: Neoplasia duration of the teaching: One month (August 2022; Number of sessions: 8	Topics: Etiological factors for neoplasia, Features of malignancy, Hallmarks of malignancy, Warburg effect, Tumour immunity, immune evasion invasion, and metastasis; Number of cartoons: 7

**Figure 2:**
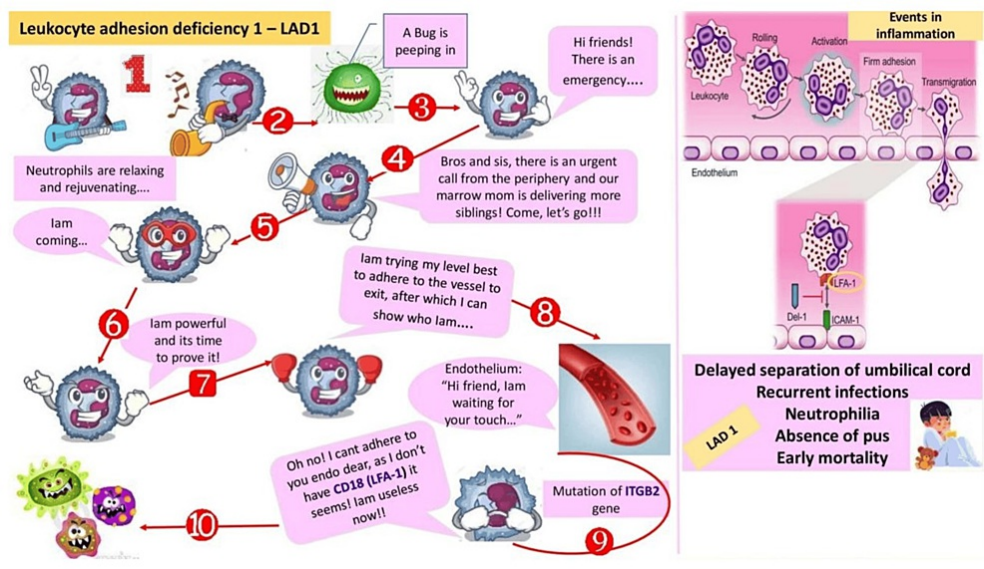
Leukocyte adhesion deficiency Cartoon by Rajeswari Kathiah

**Figure 3:**
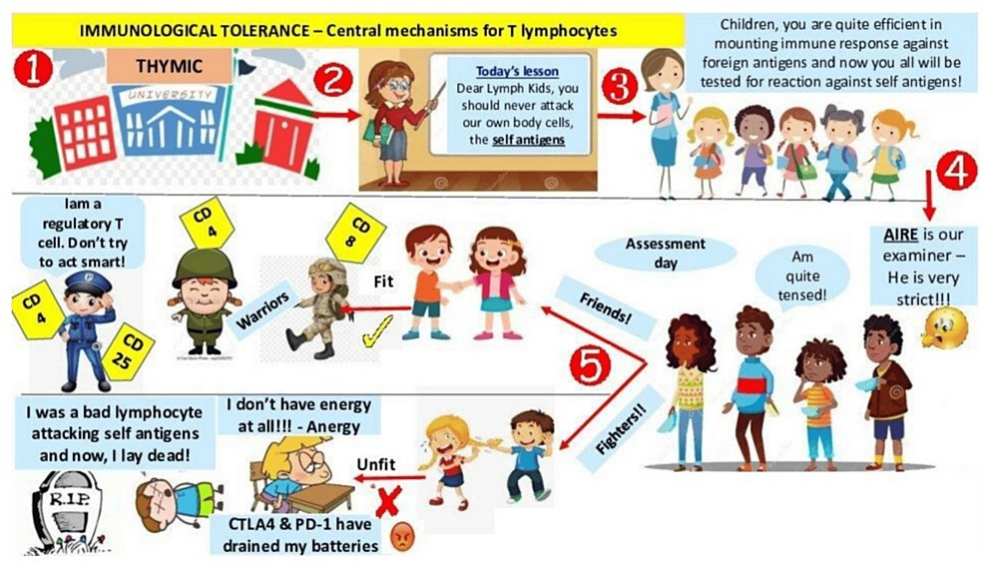
Immunological tolerance Cartoon by Rajeswari Kathiah

**Figure 4:**
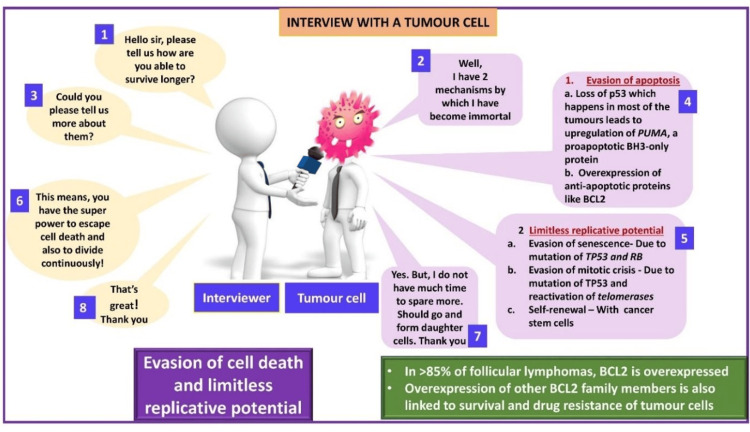
Mechanisms of evasion of cell death and limitless replicative potential of cancer cells Cartoon by Rajeswari Kathiah

**Figure 5:**
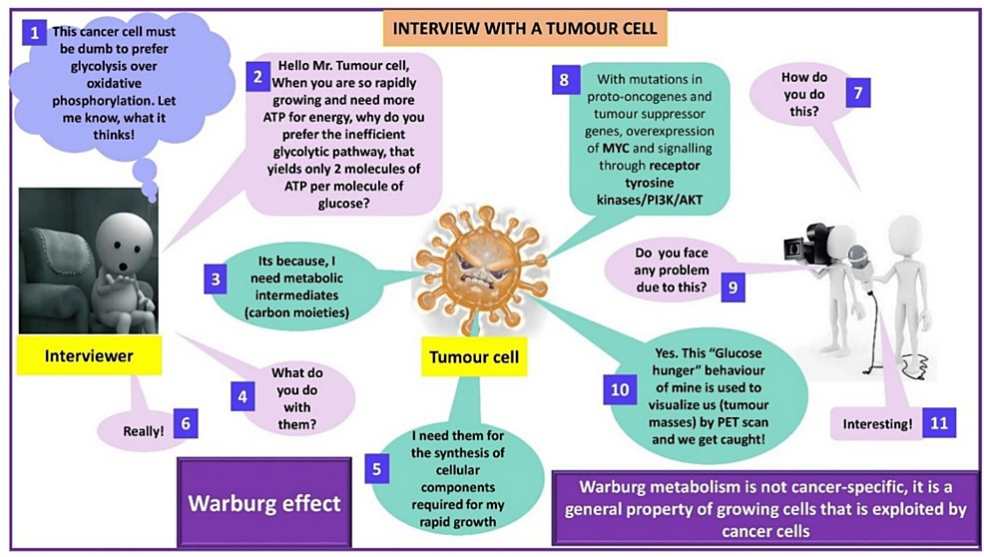
Warburg effect Cartoon by Rajeswari Kathiah

The principal investigator designed cartoons using Microsoft PowerPoint (Microsoft Corporation, Redmond, WA) with animation features and simplified complex topics for easier understanding. The cartoons show leukocyte adhesion deficiency (Figure 2), immunological tolerance (Figure 3), mechanisms of evasion of cell death and limitless replicative potential of cancer cells (Figure 4), and the Warburg effect (Figure 5). 

Group allocation and teaching methods

Using a computer-generated randomization program, students were assigned to either the "traditional group" with routine interactive teaching or the "combination group" which included both traditional teaching and cartoon-based explanations. The groups were crossed over for the second chapter to ensure equitable exposure to both teaching methods.

Assessment and feedback

Post-learning assessments were conducted one week after each chapter's completion using 50 multiple-choice questions (MCQs) formulated by the principal investigator. While the questions encompassed all topics within the chapters, the majority (75%) were centered around the more intricate subjects discussed with cartoons. Following both assessments, students anonymously completed a feedback survey consisting of 25 questions developed and validated by the research team.

Pilot study and material validation

A pilot study on 25 third-year students assessed the questionnaire’s accuracy, achieving a Cronbach’s alpha score of 0.841, thus establishing its validity and reliability. Additionally, an independent expert panel verified the face and content validity of both teaching materials (Microsoft PowerPoint presentations and cartoons), ensuring equivalence in information accuracy and content comparability.

Data analysis

Manual scoring of assessment answers was conducted, and feedback was rated on a 5-point Likert scale. The primary outcome measured was the post-learning MCQ test scores, while the secondary outcome focused on students' perceptions of the learning benefits of cartoons.

Statistical analysis

Data entry and analysis were performed using Microsoft Excel 2016 (Microsoft Corporation, Redmond, WA) and statistical software Statistical Package for Social Sciences (SPSS), version 25.0 (IBM Corp., Armonk, NY). Continuous variables were summarized as mean ± SD and categorical variables as frequency percentages. Chi-square tests with 95% CI were used for categorical variable associations. For primary outcomes, independent samples t-tests compared the post-learning test scores between groups. Chi-square tests analyzed secondary outcomes, with a two-sided p-value <0.05 considered statistically significant.

## Results

Participant demographics and study flow

Out of 109 second-year MBBS students, 106 consented to participate in the study. However, three students were absent for one or both assessments and were consequently excluded, leaving 103 students in the final analysis. This group comprised 53 (51.4%) females and 50 (48.6%) males, with an average age of 19.66 ± 0.67 years. The age and gender distribution of the participants are detailed in Table 2, showing no significant differences in these demographics among the study participants. 

**Table 2:** Age and gender distribution of study participants (N=103) * The data has been represented as N, %, (column percentages) ** P value obtained from Fisher exact test and P value<0.05 is considered statistically significant

Age in years	Females		Males		Total		P value**
	N	%*	N	%*	N	%*	
19	23	43.4	20	40	43	41.7	0.437
20	28	52.8	26	52	54	52.4	
21	0	0	3	6	3	2.9	
22	2	3.8	1	2	3	2.9	
Total	53	100	50	100	103	100	

Assessment outcomes

Students from both groups underwent assessments using MCQs after each topic. The central tendency measures of the post-learning assessment scores are depicted in Figure 1. In the combination group (interactive large-group teaching and cartoon-based teaching), the mean score following the first assessment was 38.9 ± 4.9, with scores ranging from 22 to 50. In contrast, the traditional group (interactive large group teaching only) scored a mean of 30.20 ± 5.6, with a range of 12 to 43.

Similarly, in the second assessment, the combination group maintained a high performance with a mean score of 38.7 ± 5.7 (range 23 to 50), while the traditional group scored a mean of 31.5 ± 4.9 (range 12 to 47).

Statistical analysis and significance

**Figure 6:**
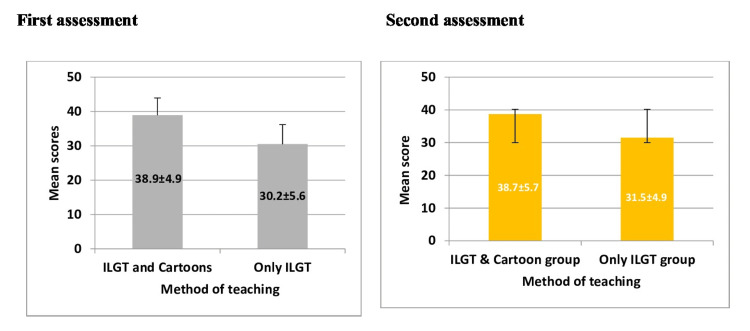
Post-learning assessment scores following the first, and second assessment ILGT: Interactive large group teaching

The independent samples t-test revealed a statistically significant difference in mean scores between the combination and traditional groups. Post-learning assessment I showed a significant advantage for the combination group (mean difference = 8.687, t(102) = 8.41, p < .001). A similar trend was observed in assessment II, with the combination group outperforming the traditional group (mean difference = 7.19, t(99) = 6.85, p < .001), as detailed in Table 3. Figure 6 depicts the post-learning assessment scores following the first, and second assessments. 

**Table 3:** Comparison of post-learning assessment scores of students * The data has been represented as mean±SD ** P value obtained from independent samples t-test and P value<0.05 is considered statistically significant

	Method of teaching	Mean±SD of scores*	t value	P value**	Mean difference	95% Confidence interval of the difference	
						Lower	Upper
I	Combination group	38.9±4.9	8.41	< .001	8.687	6.64	10.73
	Traditional group	30.2±5.6					
II	Combination group	38.7±5.7	6.85	< .001	7.19	5.11	9.27
	Traditional group	31.5±4.9					

Feedback survey results

The anonymous feedback survey, conducted after the second post-learning assessment, yielded insightful responses. A majority of students 91 (88.3%) agreed that cartoons improved their understanding of complex topics, 95 (92.2%) felt that cartoons aided in memory retention, and similar proportions believed cartoons should be extended to other complex topics in pathology and medical subjects. Furthermore, 93 (90.3%) students found cartoon-based learning to be fun and engaging. Interestingly, 95 (92.2%) participants were first-time attendees of cartoon-based teaching, and 57 (53.4%) expressed interest in creating their own educational cartoons. These responses indicate a statistically significant preference for cartoon-based teaching (χ² = 130.9, p < 0.001) (Table 4). 

**Table 4:** Responses to feedback survey on the impact of cartoons (N=103) * The data has been represented as N, %, (column percentages) ** P value obtained from Chi-square test and P value<0.05 is considered statistically significant

Items	Strongly agree N (%)*	Agree N (%)	Neutral N (%)	Disagree N (%)	Strongly disagree N (%)	X^2^ value, and P value**
Cartoons help me understand complex topics better	79 (76.7)	12 (11.6)	10 (9.7)	2 (1.9)	0 (0)	130.9 <0.001
Cartoons help me to remember complex topics better	75 (72.8)	20 (19.4)	8 (7.8)	0 (0)	0 (0)	
Cartoons should be shared for all the complex topics, and chapters of Pathology	78 (75.7)	13 (12.6)	10 (9.7)	2 (1.9)	0 (0)	
Cartoons should be shared for all the complex topics, and chapters of all the subjects in Medicine	73 (70.9)	15 (14.6)	9 (8.7)	5 (4.9)	1 (1)	
Cartoons help me to have “Fun learning”	80 (77.7)	13 (12.6)	10 (9.7)	0 (0)	0 (0)	
I attended cartoon-based teaching, and reinforcement of concepts for the first time	95 (92.2)	0 (0)	0 (0)	0 (0)	8 (7.8)	
Would try to make my own cartoons for my understanding in the future	25 (24.3)	30 (29.1)	18 (17.5)	17 (16.5)	13 (12.6)	

## Discussion

Traditional didactic lectures have long been the cornerstone of medical education, often characterized by instructor-led delivery of substantial information with limited student interaction. These lectures, typically conducted in an instructor-centric environment, centralize knowledge and content but may lack significant student involvement [3,4]. However, learning is fundamentally an active process requiring collaboration between teachers and learners to make the educational experience enjoyable and effective [5,6].

For learning to be truly effective, students should be able to apply knowledge and skills acquired in the classroom toward their professional aspirations. This necessitates a diverse range of learning styles, opportunities for feedback, and discussions that enhance learning effectiveness [6,7]. Thus, the shift towards innovative teaching methods, supplementing didactic lectures with interactive large-group teaching, is gaining momentum. When complemented with creative educational tools, interactive teaching not only enhances learning but also improves retention and makes the process more enjoyable [8-10].

Recent trends in medical education lean towards augmenting traditional or interactive teaching methods with imaginative educational materials. Cartoons, or graphic stories, effectively combine images and text, providing visual cues that align with the “dual-coding model of multimedia learning.” This model suggests that learning is enhanced when materials are presented both verbally and visually in a sequential and coordinated manner [11,12]. Cartoons have a unique ability to communicate information engagingly, using symbols and imagery to captivate learners and enrich their educational experience. The emotional engagement with cartoon characters and storylines can motivate students, potentially aiding in memorization and sustained interest [13-15].

Our study supports the positive influence of cartoons as supplementary educational tools. The significant improvement in class averages for students taught with cartoons, and the feedback survey results, indicate that students find cartoons helpful in understanding and remembering complex topics. The majority reported enjoying cartoon-based teaching, noting its efficacy in concept comprehension and retention.

To our knowledge, this is the first experimental study done to evaluate the effectiveness of cartoon-based teaching in medical education, specifically in the challenging field of pathology. By randomly allocating students to intervention and control groups, our study minimized selection and confounding biases. Recognizing that self-study can be influenced by various external factors, we conducted a pilot study in a controlled classroom setting to ensure the effectiveness of our teaching approach before implementing cartoons in the main study.

Our findings suggest that traditional lecture methods might not suit every medical student's learning style. This study introduces a novel and positive approach to utilizing cartoon materials in teaching, potentially transferring core medical knowledge as effectively, if not more so, than traditional methods [16-18] Embracing innovative teaching approaches could lead to enhanced outcomes for current and future medical students. Creating quality cartoon content may be challenging, but the collaboration between medical educators and artists can yield fruitful results. Our experience shows that cartoon-based teaching not only facilitates learning but also inspires students to create their educational materials, further reinforcing their grasp of the subject matter [19].

Limitations

While our study provides valuable insights, it is important to acknowledge its limitations

Sample Size and Duration

The relatively small sample size and brief duration of the study may limit the generalizability of our findings. Future studies with larger cohorts and extended timelines are essential to comprehensively evaluate the long-term effectiveness and retention benefits of cartoon-based learning.

Cartoon Preparation

The creation of cartoons by individual faculty members poses a significant challenge. This limitation could be mitigated by collaborative efforts within departments to pool resources or by utilizing copyright-free cartoons available online, thus ensuring a wider variety and accessibility of educational materials.

Learning Styles and Cultural Impact

The study did not account for the influence of students' preferred learning styles or the impact of diverse geographic learning cultures. These factors could play a critical role in the effectiveness of cartoon-based teaching and should be considered in future research.

Style and Character of Cartoons

Our study does not address which specific styles or characters in cartoons are most effective for educational purposes. Identifying the most impactful types of cartoon imagery and narratives could further enhance the effectiveness of this teaching method.

Addressing these limitations in subsequent research will be crucial to fully understanding the potential and scope of cartoon-based teaching in medical education.

## Conclusions

The findings of our study underscore the significant advantages of integrating cartoons into traditional teaching methods. This innovative approach not only captivates students' attention more effectively but also leads to superior post-learning test scores compared to conventional teaching alone. Our results advocate for the inclusion of cartoon-based teaching in medical education, offering a novel and engaging way to enhance learning outcomes. We anticipate that our study will inspire medical educators worldwide to explore the use of cartoons and conduct further research into their effectiveness and appeal among students, potentially transforming the landscape of medical education.
